# Featuring the Features of the Featureless Tremor: A Statement about Essential Tremor

**DOI:** 10.5334/tohm.931

**Published:** 2024-07-26

**Authors:** Elan D. Louis

**Affiliations:** 1 Department of Neurology, University of Texas Southwestern Medical Center, Dallas, Texas, USA; 2 Peter O’Donnell Jr. Brain Institute, University of Texas Southwestern Medical Center, Dallas, Texas, USA

**Keywords:** essential tremor, diagnosis, clinical, classification, examination

Earlier this month, Luo et al. published a noteworthy article in *Tremor and Other Hyperkinetic Movements*, focusing on the clinical semiology of tremor in a patient with Spinocerebellar Ataxia (SCA) 12 [1]. Early in the course of SCA 12, tremor can be the primary, and in some instances the only, clinical feature, and patients may be misdiagnosed as essential tremor (ET) [2,3,4]. However, as demonstrated by Luo et al., [1] the clinical features of their patient, when carefully assessed, were at variance with what has been described in patients with ET. On one level, their work adds to our understanding of the features of tremor in patients with SCA 12 through a careful description of the neurological examination findings in their patient. On a broader level, their work underscores the fact that ET is an over-diagnosed condition, and this is due to a failure to recognize its clinical nuances.

The clinical features of ET have often been oversimplified, with many viewing an isolated, non-specific action tremor as the sole manifestation. However, ET presents with a variety of nuanced features that require careful assessment. It was not uncommon in the published literature for the disease itself to be viewed merely as a physical examination finding. A consensus statement on the classification of tremor, published in 2018, which is widely-read and cited, simply described the tremor in ET as “bilateral upper limb action tremor” [5].

Aside from the clear presence of dystonia [6], ataxia [7], cognitive impairment [8] and rest tremor [9] in patients with more advanced ET, in recent years, there is a considerable literature on the many and nuanced features of the action tremor itself in ET [10]. As such, the tremor of ET is neither non-descript nor featureless but rather, is multifaceted and highly patterned. Among these described features are: the centrality of kinetic tremor as the key feature; the greater amplitude of kinetic than postural tremor; the tendency for the tremor to involve the wrist and elbow rather than metacarpophalangeal joints; the observation that the wrist tremor more often involves a flexion-extension than a pronation-supination movement; the presence of rest tremor, restricted to the upper limbs, in advanced cases; and the observations that in many patients, the tremor waveforms align along a single predominant axis during spiral drawing, the neck tremor generally resolves when the patient is supine, and the jaw tremor more commonly occurs when the mouth is open. As such, the action tremor in patients with ET is not simply *any* upper limb action tremor [10].

That the tremor in ET is treated as featureless is evidenced by the observation, in several studies, that up to 55% of individuals who receive an “ET” diagnosis do not have ET [11,12,13]. Rather, they have dystonia with dystonic tremor, Parkinson’s disease or other diseases. A failure on the part of the diagnosing physician to recognize these features is at the heart of this problem.

It is not only in clinical care situations that an incorrect diagnosis is problematic. In research studies, the issue of diagnostic misclassification can lead to false null results (i.e., a type II error), as the supposed cases of “ET” have other diagnoses rather than ET. At the heart of this is superficial phenotyping [14,15].

Physicians, particularly neurologists, must deepen their understanding of ET, starting with a comprehensive appreciation of its clinical features. This is particularly important, as the diagnosis of ET during life is dependent on the neurological examination, as there are no biological diagnostic markers for ET. In sum, as reflected in the manuscript by Luo et al., [1] it is important to appreciate the features of the “featureless tremor”.
